# A Virtual Community for Disability Advocacy: Development of a Searchable Artificial Intelligence–Supported Platform

**DOI:** 10.2196/33335

**Published:** 2021-11-05

**Authors:** Christo El Morr, Pierre Maret, Fabrice Muhlenbach, Dhayananth Dharmalingam, Rediet Tadesse, Alexandra Creighton, Bushra Kundi, Alexis Buettgen, Thumeka Mgwigwi, Serban Dinca-Panaitescu, Enakshi Dua, Rachel Gorman

**Affiliations:** 1 School of Health Policy and Management Faculty of Health York University Toronto, ON Canada; 2 CNRS, UMR 5516, Laboratoire Hubert Curien, Université Jean Monnet Saint Etienne Saint Etienne France; 3 Student Learning and Academic Success Department, York University Libraries York University Toronto, ON Canada; 4 School of Gender, Sexuality and Women's Studies York University Toronto, ON Canada

**Keywords:** virtual community, machine learning, Semantic Web, natural language processing, web intelligence, health informatics, Wikibase, disability rights, human rights, CRPD, equity, community, disability, ethics, rights, pilot, platform

## Abstract

**Background:**

The lack of availability of disability data has been identified as a major challenge hindering continuous disability equity monitoring. It is important to develop a platform that enables searching for disability data to expose systemic discrimination and social exclusion, which increase vulnerability to inequitable social conditions.

**Objective:**

Our project aims to create an accessible and multilingual pilot disability website that structures and integrates data about people with disabilities and provides data for national and international disability advocacy communities. The platform will be endowed with a document upload function with hybrid (automated and manual) paragraph tagging, while the querying function will involve an intelligent natural language search in the supported languages.

**Methods:**

We have designed and implemented a virtual community platform using Wikibase, Semantic Web, machine learning, and web programming tools to enable disability communities to upload and search for disability documents. The platform data model is based on an ontology we have designed following the United Nations Convention on the Rights of Persons with Disabilities (CRPD). The virtual community facilitates the uploading and sharing of validated information, and supports disability rights advocacy by enabling dissemination of knowledge.

**Results:**

Using health informatics and artificial intelligence techniques (namely Semantic Web, machine learning, and natural language processing techniques), we were able to develop a pilot virtual community that supports disability rights advocacy by facilitating uploading, sharing, and accessing disability data. The system consists of a website on top of a Wikibase (a Semantic Web–based datastore). The virtual community accepts 4 types of users: information producers, information consumers, validators, and administrators. The virtual community enables the uploading of documents, semiautomatic tagging of their paragraphs with meaningful keywords, and validation of the process before uploading the data to the disability Wikibase. Once uploaded, public users (information consumers) can perform a semantic search using an intelligent and multilingual search engine (QAnswer). Further enhancements of the platform are planned.

**Conclusions:**

The platform ontology is flexible and can accommodate advocacy reports and disability policy and legislation from specific jurisdictions, which can be accessed in relation to the CRPD articles. The platform ontology can be expanded to fit international contexts. The virtual community supports information upload and search. Semiautomatic tagging and intelligent multilingual semantic search using natural language are enabled using artificial intelligence techniques, namely Semantic Web, machine learning, and natural language processing.

## Introduction

Human rights monitoring for people with disabilities is a pressing issue in Canada and France, as well as internationally. However, data and information about human rights policy and legal precedents, lived experiences, and media portrayals of people with disabilities are scarce. This limited data and information hinders the capacity for knowledge mobilization among researchers, nongovernmental organizations, and advocacy groups in their efforts to monitor the implementation and realization of human rights for people with disabilities. In addition, policy-based definitions of disability vary widely across local, national, and international jurisdictions, creating further challenges regarding the access of comparative data and information.

Internationally, a few sources provide access to some disability data (eg, the Disability Data Advocacy Working Group [1], Disability Data Portal [2], and Disability Rights Promotion International [DRPI] [3]). However, these databases have limited data while the need for such data is overwhelming. The incorporation of disability into human rights–focused legislations, constitutions, and international law (eg, the United Nations Convention on the Rights of Persons with Disabilities, or CRPD [4]) has highlighted an ongoing need for access to disability data for disability researchers, organizations of people with disabilities, and broader public policy and development sectors. The lack of availability of disability data has been identified as a major challenge for continuous disability rights monitoring (ie, collection, integration, analysis, interpretation, and mobilization of data and knowledge about the implementation and realization of human rights for people with disabilities) [5,6]. It is important to develop an inclusive approach to analytics and machine learning, one that can be scaled up to integrate disability data and enable the searching of such data to expose systemic discrimination and social exclusion, which increase vulnerability to inequitable social conditions [7].

Historically, there has been a lack of data on disability collected and shared on a global and national level [8]. The lack of aggregate and disaggregate disability data available is hampering researchers’ ability to conduct critical health research [9]. It is also limiting our knowledge about the experiences of people with disabilities and the challenges and barriers they face [6]. Reed, Meeks, and Swenor [10] argue that “the disability data gap is more than just a surveillance oversight; social injustices exist that cannot be separated from this lack of information. The lack of data perpetuates the exclusion of disabled people from discussions of health equity and policies that are data driven.”

Disability is a complex multidimensional experience that can be difficult to measure. Operational measures of disability vary according to the purpose and application of the data, the conception of disability, and the aspects of disability examined [10]. However, access to disability data is an issue of human rights. For example, Article 31 of the CRPD [11] notes the following obligation:

States Parties undertake to collect appropriate information, including statistical and research data, to enable them to formulate and implement policies to give effect to the present Convention… The information collected shall be disaggregated, as appropriate, and… States Parties shall assume responsibility for the dissemination of these statistics and ensure their accessibility to persons with disabilities and others.

The disability Wikibase addresses this responsibility by making data accessible and available to a variety of stakeholders. Understanding, interpreting, and using disability data is essential to stakeholders with interests in disability issues, including advocacy groups, individuals, and organizations of people with disabilities, nongovernmental organizations, policy makers, and researchers [12]. Data contribute to decisions about poverty alleviation strategies, health and social service development, and humanitarian responses. Data collection is critical to the formulation of evidence-based policies and all aspects of the implementation of disability-inclusive policies and programs [13]. Reliable, accessible data are also crucial for monitoring progress and implementation of the CRPD.

Meanwhile, advancements are being made in the field of health informatics, which can assist in the organization and use of health data. Health informatics is an interdisciplinary field that uses computer science, decision-making, information processing, and management science to design and implement information systems in a user-centered manner to get, store, process, and provide information for users to assist them in achieving their objectives [14]. Some of the techniques used in health informatics are artificial intelligence (AI) techniques [15]. Machine learning techniques are trained algorithms that assist users in making sense of data to make organizational decisions [16] and health decisions [17,18]. Semantic Web techniques and natural language processing (NLP) provide computers with the ability to decipher the meaning of information and process phrases written or spoken in natural language [19]. The combination of these techniques can allow users to search for data using natural language sentences [20,21] in multiple languages, and they can also be used to find semantic similarities among disparate documents from different disciplines [22-26].

On the other hand, techniques to store, integrate, and search for data do exist; the publicly available Wikibase environment is an open-source collaborative tool for editing, integrating, and storing structured data. Wikidata is the most famous Wikibase example, and it targets public knowledge [27].

Our project aims to create a screen reader–accessible multilingual website on top of a Wikibase that structures and integrates data about people with disabilities and serves national and international disability advocacy communities. This pilot website will be endowed with a document upload function with an automated data-from-text extraction function for insertion into the Wikibase, and with an intelligent natural language search capability for querying the data.

## Methods

### Choice of Domain and Documents

To design and develop the underlying data model of the pilot platform, our domain of choice was the CRPD. The CRPD is an international human rights treaty intended to articulate and protect the rights and dignity of people with disabilities. The CRPD reflects a shift from viewing people with disabilities as objects of charity, medical treatment, and social protection toward viewing them as full and equal members of society with human rights. The CRPD contains 50 Articles covering a range of issues including health, accessibility, data collection, and monitoring mechanisms and is informed by the principles of dignity, autonomy, nondiscrimination, equality, and respect for difference. The CRPD has been signed by over 150 countries, including Canada and France.

This domain of choice allows for a large number of documents connected to the CRPD to be uploaded in the future, enabling the testing of the platform on a large scale. Our first set of documents to be shared included DRPI’s reports of country- and region-specific rights monitoring based on the CRPD. DRPI is a collaborative project to establish a comprehensive, sustainable international system to monitor the human rights of people with disabilities in accordance with the CRPD. This project has been operating for over a decade and has collected data and information from North America, Europe, Latin America, Asia Pacific, Africa, the Middle East, and North Africa.

### Platform Design

In early stages of the project, we collected the requirements of the platform and identified that the platform should act as a virtual community and incorporate the following characteristics:

1. Facilitate knowledge sharing by enabling the uploading of disability documents.
2. Provide validated information by allowing only authenticated external partners (eg, community organizations) to add documents and having a user approve them, acting as a gatekeeper.
3. Support disability rights advocacy by enabling dissemination of knowledge to the public by making the documents available to all potential users and using a natural language–enabled search engine.
4. Administer the platform to create users, continuously refine the machine learning models, provide reports, etc.

In summary, the aim of this project was to create a virtual knowledge network using the Semantic Web and other AI techniques. The virtual knowledge network includes 4 types of users:

1. Authenticated information producers that upload information of interest (eg, reports, blogs, articles) and assign tags (ie, keywords) to the new data.
2. Domain expert users that play the role of gatekeepers and validate the uploaded information (ie, documents and tags) by issuing an upload request to the platform administrator.
3. Platform administrators that manage the platform. Their tasks include approving/rejecting upload requests, continuously maintaining the platform, and finetuning the AI models.
4. Information consumers that search for information stored on the platform using natural language questions.

### Platform Implementation

The virtual knowledge network front end includes a simple website connected to a search engine, while the back end uses the following:

1. A machine learning model to tag each paragraph of the uploaded documents with corresponding keywords that reflect the meanings embedded in the text.
2. A Wikibase structure that holds the disability rights knowledge base and the corresponding data (ie, tags, annotated paragraphs, links to the documents).
3. An intelligent search engine (QAnswer) that uses NLP to search the Wikibase for answers to users’ questions.

To implement the different parts of the platform, the following major programming stacks were used:

1. React JS, a simple and flexible JavaScript-based library, was used to develop the front end of the platform [28].
2. MySQL was our choice for the database used to store temporary document information before its semantification and storage in the Wikibase [29].
3. Wikibase was used to store the disability rights knowledge base, which was represented in a knowledge graph and used the Resource Description Framework (RDF) format [30]. A knowledge graph is a network of entities, their semantic types and properties, and the relationships between the entities [31]. It is used to represent information and reasoning.
4. QAnswer, a platform that makes RDF data accessible via natural language, has been used to enable intelligent search using intelligent natural language questioning [32-34]. QAnswer is the first AI-driven platform to query RDF datastores in natural language. It uses semantic technologies (NLP, Semantic Web) as well as machine learning techniques [32].
5. Python was used to develop cross-platform and server-side applications [35].
6. FastText was used for NLP to create a machine learning model to tag paragraphs. FastText is often on par with deep learning classifiers in terms of accuracy, and is many orders of magnitude faster for training and evaluation [36,37].
7. PyMuPDF, a Python library, was used to access and process uploaded documents [38].
8. GitHub was used to maintain source code versioning [39].

## Results

### Disability Data Modeling

#### CRPD Ontology

**Figure 1:**
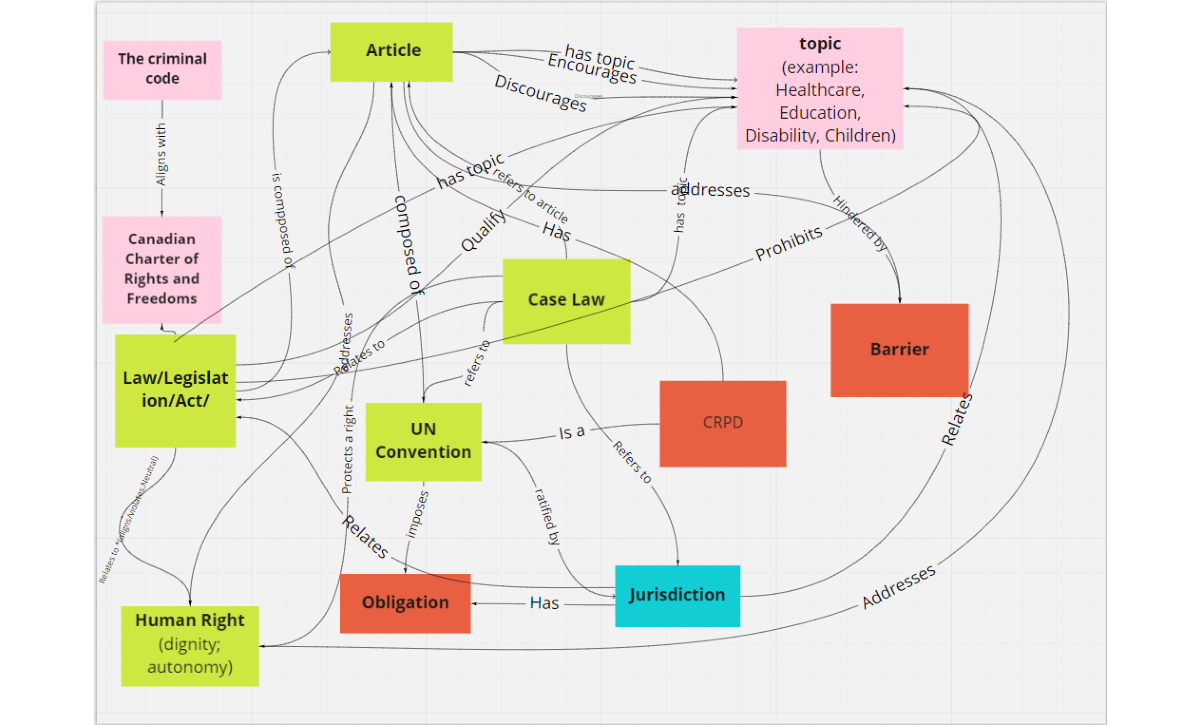
CRPD ontology. CRPD: Convention on the Rights of Persons with Disabilities; UN: United Nations.

Data modeling includes identifying the correct ontology for storing document data and finding appropriate relations between different data entities to map the data correctly within the Wikibase. Our team developed an ontology schema (Figure 1) for the CRPD that is flexible and can accommodate the CRPD Articles as well as incorporate jurisdiction-specific legislation so that users will be able to tag and search disability-related information in relation to internationally and locally recognized rights domains.

#### CRPD Knowledge Graph

Based on the ontology, domain experts developed structured data that describes the CRPD document. The structured data (Table 1) was used to create the CPRD knowledge graph.

**Figure 2:**
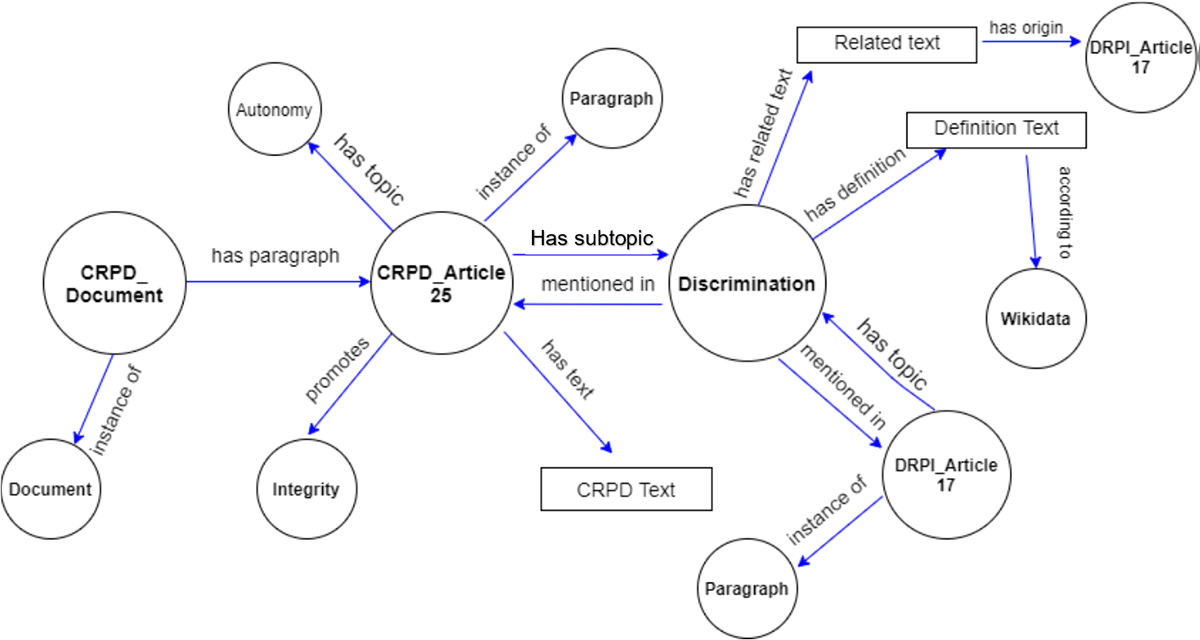
A partial knowledge graph that depicts entities and relationships present in CRPD Article 25. CRPD: Convention on the Rights of Persons with Disabilities; DRPI: Disability Rights Promotion International.

The structured data was used to develop a detailed knowledge graph that describes the CRPD; for instance, Table 1 shows that Article 25 of the CRPD relates to the topic “Health.” The knowledge graph (Figure 2) shows the same information as a graph. The knowledge graph is practically a set of triples (ie, subject-predicate-object) and was implemented in the disability Wikibase, which is a datastore that stores triples. Once incorporated in the disability Wikibase, the knowledge graph allowed semantification of the DRPI documents into triples (eg, document - has paragraph - the paragraph text; paragraph - has topic - discrimination). Semantification allows for a semantic search to be performed on the documents and their paragraphs.

**Table 1:** Structured data prepared by domain experts to develop the Convention on the Rights of Persons with Disabilities knowledge graph.

Article 25 (Health) topics	Has-topic	Text
Health	has-CRDP-Text	*Provide persons with disabilities with the same range, quality, and standard of free or affordable health care and programs as provided to other persons, including in the area of sexual and reproductive health and population-based public health programmes*
Discrimination	Mentioned-In-CRPD-Article	*Prohibit discrimination against persons with disabilities in the provision of health insurance, and life insurance where such insurance is permitted by national law, which shall be provided in a fair and reasonable manner*
Preventative measures	has-CRDP-Text	*Provide those health services needed by persons with disabilities specifically because of their disabilities, including early identification and intervention as appropriate, and services designed to minimize and prevent further disabilities, including among children and older persons*

Figure 2 shows the relationships among the entities of articles 25 and 17 of the CRPD using a knowledge graph. Documents, topics, and sources are concepts connected by relations (eg, “has topic,” “has paragraph,” “according to,” “mentioned in,” “has text,” “has definition,” “has origin,” “instance of”). For instance, paragraphs are connected to the document using the “has paragraph” relation since each document is formed of many paragraphs. In turn, each paragraph has one or more topics and subtopics, and so on.

### Topic Modeling Using Latent Dirichlet Allocation

We used a latent Dirichlet allocation (LDA) [40] machine learning algorithm to model the topics in the available documents. To train the model, a set of 30 unstructured and unlabeled documents were provided in an unsupervised manner. To determine the number of topics to include in this unsupervised learning method, we conducted tests to select the best coherence measure score; this score is based on the degree of semantic similarity between high-scoring words in the topic.

The LDA result provided disability experts with an initial list of keywords that could be used to tag the documents’ content. The disability experts added to the list of keywords and created a glossary of terms related to the disability field. The glossary terms were used as labels for tagging the paragraphs of a training set of the document corpus. Afterward, the labeled documents were used by the supervised machine learning algorithm proposed by the FastText library to design a text classification model that allows users to tag uploaded documents.

### Initial Glossary Development and Enrichment

Disability domain experts developed a glossary of keywords that describe the main topics and subtopics embedded in the DRPI and CRPD documents. These keywords were then used to tag paragraphs of the uploaded documents with appropriate topics.

Following the development of the glossary, the Merriam-Webster Dictionary application programming interface (API) was used to get synonyms of the glossary terms. Thus, the glossary terms were enriched with synonym words.

### Document Tagging

#### Overview

**Figure 3:**
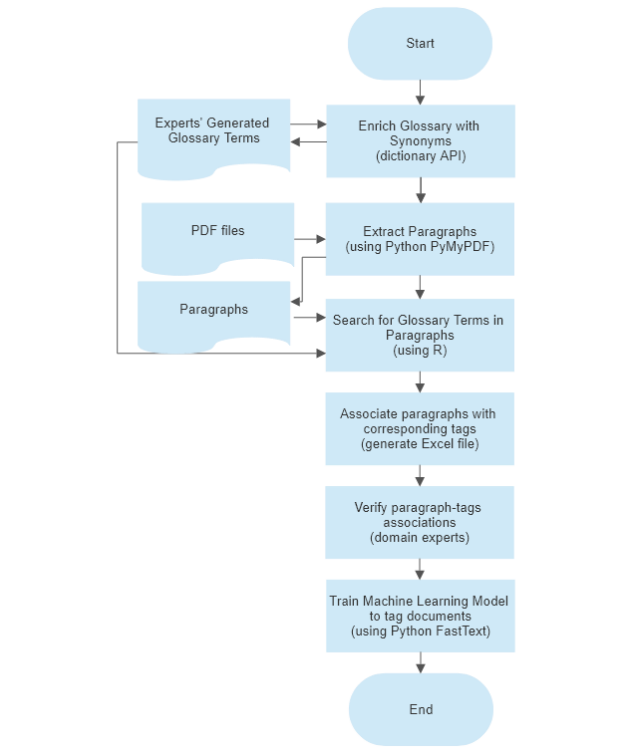
Document tagging flowchart. API: application programming interface.

Once a user uploads a document, the platform splits the document into paragraphs and uses the glossary terms to tag them with appropriate topics. In the first phase, the extracted paragraphs and corresponding tags were verified by domain experts and the result was used as a training data set for a machine learning algorithm (Figure 3). The algorithm was used on the platform to automatically tag newly uploaded documents.

#### Paragraph Extraction and Tagging

Using the PyMuPDF library (a Python binding for MuPDF), the PDF files were parsed, and paragraphs were extracted. Afterward, R (version 4.0.5; R Foundation for Statistical Computing) was used to tag the paragraphs with appropriate glossary terms. The tags were reviewed by domain experts who added or deleted tags as needed. The result was a set of validated instances of paragraphs and their corresponding tags, which were used as the training data set.

#### Machine Learning Model Generation to Automate Tagging

The validated paragraph-tag instances were used as a training set for the FastText algorithm. FastText is a library for efficient learning of word representations and sentence classification. This library can be used to build a word representation model with unsupervised training (using SkipGram or CBOW), or to design a text classification model with supervised learning. We used the latter approach and used the FastText library defaults for tagging the document paragraphs.

**Figure 4:**
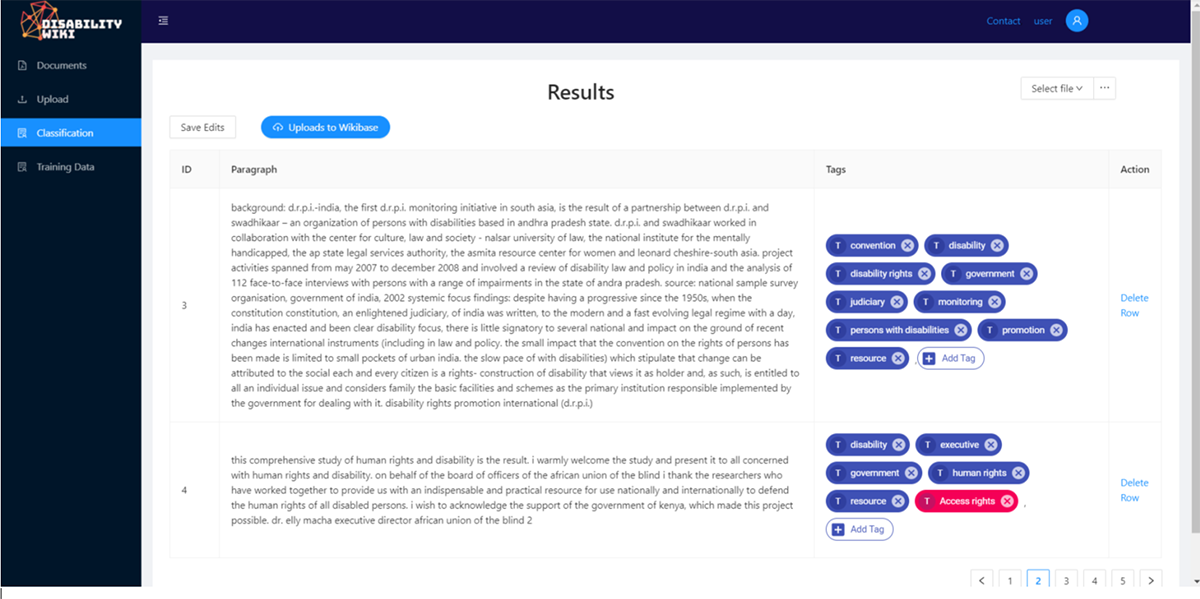
Paragraphs and their tags are displayed to the user.

The FastText algorithm proposes tags from the glossary for the paragraphs and the result is displayed (Figure 4). Users can reject or add to the proposed tags, allowing for continuous enrichment of the glossary and training of the model, which make it more accurate. A request to upload the tagged paragraphs and other related information to the Wikibase is sent to the administrator.

### Document Upload to Wikidata

**Figure 5:**
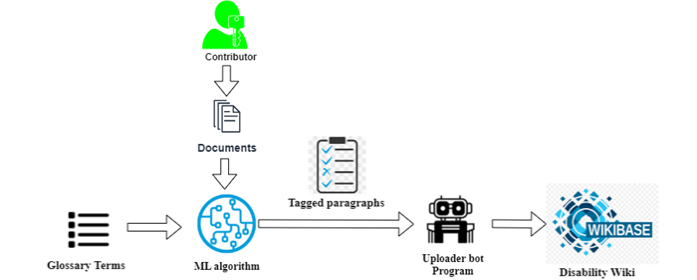
Architecture diagram for the document upload process flow. ML: machine learning.

The administrator can review the upload requests and accept or reject them. Upon acceptance, the tagged paragraphs are semantified based on the ontology and uploaded to the disability Wikibase, where they are stored as triples that can be subject to semantic search (Figure 5).

### Natural Language Search Using QAnswer

**Figure 6:**
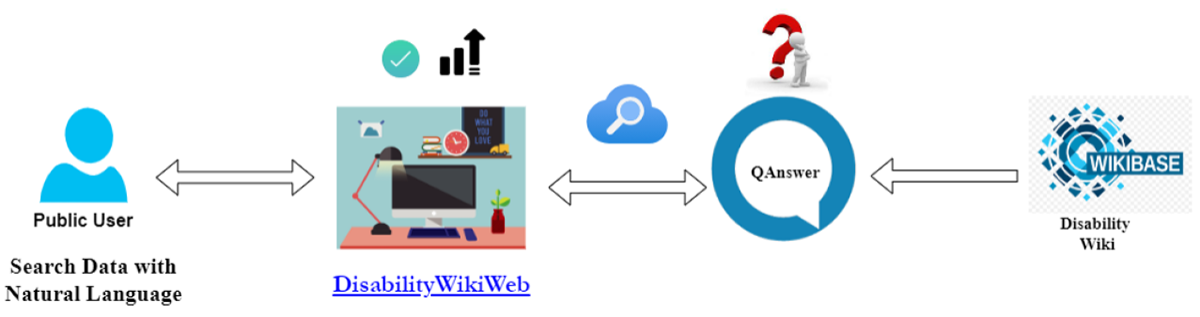
Search function flow of the disability wiki website.

The disability Wikibase is connected to the QAnswer search engine, which performs a natural language search (Figure 6). A simple search text field on the website is provided for users to enter their queries.

Although only authorized users can be producers and upload documents, any user can be a consumer and search for information. The disability Wikibase uses QAnswer API endpoints to send questions to, and retrieve answers from, QAnswer. We used the autocomplete feature in QAnswer to provide several precompleted questions while users are typing their questions. Thus, users can select from these autocompleted options instead of continuing to type their question.

## Discussion

### Principal Results

There are currently no tools available to collect and share data related to people with disabilities. We have developed a platform that can fill this gap and respond to this need.

Using health informatics and AI techniques, we were able to develop a pilot virtual community that supports disability rights advocacy by facilitating the sharing and accessing of disability data. The virtual community accepts 4 types of users: information producers, information consumers, validators, and administrators. The virtual community enables the uploading of documents, semiautomatic tagging of document paragraphs with meaningful keywords, and validation of the process before the document and its associated tags are uploaded to the disability Wikibase. Once uploaded, public users can perform a semantic search using the QAnswer intelligent search engine. Currently, the platform allows information producers to upload documents and label their paragraphs with appropriate tags, and also allows information consumers to search for information using natural language. Hence, the platform answers a serious need in the disability community to store disability-related data in one database that includes an intelligent search function.

Virtual communities for health purposes (including disabilities) already exist; however, their purpose is to disseminate information only, with no means for members to contribute additional data and information. Wikipedia is wiki-based but it is not specialized and includes nonvalidated, general-purpose information, with no means to upload documents or perform a semantic search. This is the first time an AI approach was used to bolster disability advocacy. The proposed knowledge graph’s content has been validated by disability domain experts, and it is also technically sound—Wikidata’s best practices were followed. Future evolutions of the knowledge graph will have to maintain its accuracy, consistency, and conciseness, as defined by Zaveri et al [41].

This project advances our previous informatics work on a virtual knowledge network using documents from the DRPI [42]; in our previous work, we developed a virtual knowledge network to share documents [42], while on this pilot site we enabled semantic search and allowed accurate tagging of documents’ content using machine learning approaches. The current platform simplifies document sharing and offers the public, disability organizations, researchers, and policy makers a tool to allow for more transparency in disability data.

Our proposed generic novel approach is to provide a platform for semantic free-text search on freely collected documents on a given topic. Our future research aims to answer the following question: what kind of information is searched for and by what types of users? To answer this question, we are planning to collect information regarding the users of the platform and the search terms they use; in addition, a thematic analysis will also be conducted to understand the users’ needs.

### Limitations and Future Perspectives

This work is still in its pilot stage; there is a limited number of documents available for machine learning training, while accuracy measurement and evaluation of the machine learning algorithms are underway. However, the more users upload documents and validate the automatic tagging, the more accurate the automatic tagging becomes due to continuous retraining.

There are other features and project phases planned for the future. Virtual communities have been used in many domains, including health care [43], to address chronic disease management, education [44], and mental health. Our current platform is expanding the use of virtual communities for social purposes in the domain of disability rights [45]. The next phases of the project will involve an evaluation of the adoption of this virtual community using evaluation frameworks [46], as well the use of background analytics to analyze usage patterns and enhance the search capacity. Moreover, users’ experience will be formally evaluated to enhance acceptance and adherence. Universal accessibility allows equitable access to information; accessibility testing is underway, and improvements are being implemented. Additionally, while QAnswer can answer questions formulated in English and French, the current platform is written in English; translating the website into French and uploading documents written in French are both on our project agenda.

NLP has been used for many health care applications for information extraction, sentiment analysis, search extraction, and information synthesis [47], using the state-of-the-art NLP model Bidirectional Encoder Representations from Transformers (BERT) [48]. Our next steps include tagging optimization using newer approaches to NLP such as BERT. This platform may encourage the creation of a domain-specific DisabilityBERT, similar to the BioBERT adapted for biomedical text and SciBERT adapted for science text [49].

In our future work, we will continue to elucidate biases within the disability Wikibase search. Although data analytics is important for collecting evidence and making decisions [50,51], neither machine learning nor data are value-free [52]. Deciding which data items will be collected is a process that embeds a worldview (Weltanschauung). For example, is the information that is collected and searched about “abnormality” [53] or “participation restriction” [54]? In addition, the production of information is done for a certain purpose and hence is not value-free; for example, were certain data collected to understand the efficiency of people with disabilities in doing a task, or to determine if people with disabilities face discrimination in schools, universities, or the workplace? In this way, our virtual community is informed and designed in accordance with the human rights principles of the CRPD. Finally, the computer algorithms are value-loaded; indeed, predictive machine learning algorithms “learn” (ie, create models) from past data to predict events in the future based on current data. However, past data embeds values and biases from the past; for example, in 2018, Amazon’s AI-based recruiting tool was found to be sexist as it consistently suggested that male applicants be recruited [55], as it learned from resumes previously submitted to the company, which is in the male-dominated tech industry. Our choice to use the CRPD to build our ontology represents a first step in creating a search algorithm for disability data to be used by and for disability advocates and communities.

The current project requires many improvements and feature enhancements. First, the document data is semantified using a few relations, such as “has paragraph,” “has topic,” and “mentioned in.” Future work will include other relations, such as “comply with,” “supports,” and “has legislation” and update the machine learning classifier to extract such semantic information from the uploaded document. Uploaded documents could be linked to their corresponding country as some documents are country- and region-specific. The current version of the application contains such properties in the interface; however, it is not embedded in the Wikibase data model. In the next phase, it is important to semantically link documents with countries and regions by importing countries and regions from Wikidata to the disability Wikibase.

Finally, the web application should be updated to include message broker architecture that allows server-side load balancing to handle large volumes of search requests and optimize the server’s performance.

### Conclusions

The platform ontology was based on the CRPD to create a platform for equity-focused disability data that can be used by and for disability advocates and communities; the data includes advocacy reports and disability policy and legislation from specific jurisdictions or international contexts. The virtual community accepts 4 types of users: information producers, information consumers, validators, and administrators. Semiautomatic tagging and intelligent semantic search using natural language are enabled using AI techniques. The search engine supports bilingual (English and French) search and further enhancements are planned.

Multidisciplinary research in the domain of disabilities is challenging. An iterative process completed in a collaborative atmosphere allows team members to elucidate ambiguities and helps the team face challenges and provide solutions.

## Abbreviations

AI: artificial intelligence
API: application programming interface
BERT: Bidirectional Encoder Representations from Transformers
CRPD: Convention on the Rights of Persons with Disabilities
DRPI: Disability Rights Promotion International
LDA: latent Dirichlet allocation
NLP: natural language processing
RDF: Resource Description Framework
